# Nonlinear Dynamics and Entropy of Complex Systems: Advances and Perspectives

**DOI:** 10.3390/e24081014

**Published:** 2022-07-22

**Authors:** Jiri Petrzela

**Affiliations:** Department of Radio Electronics, Faculty of Electrical Engineering and Communication, Brno University of Technology, Technicka 12, 616 00 Brno, Czech Republic; petrzela@vut.cz

Biological, engineering, economic, social, medical, environmental, and other systems exhibit time evolution. These observable objects can be either naturally inspired or artificial, isolated or non-autonomous, and truly deterministic or stochastic. Some of them can be, after appropriately executed simplification, modelled by suitable mathematical expressions, usually in the form of ordinary differential equations. Since global models need to be treated as nonlinear, complicated types of dynamical behavior that include multistability and chaos represent possible solutions.

In addition to its application in information theory, entropy is a general measure frequently used for the qualitative analysis of complex systems. Aside from its mathematical definition, entropy describes the complexity of dynamics with respect to internal system parameters, initial conditions, external forcing, time instances, or other factors that affect system dynamics.

Using our knowledge of mathematical models, powerful modern personal computers can be used to localize specific types of dynamic behaviors. For example, Petrzela [1] investigated a fundamental single-stage amplifier with resonant load where the only active element is a bipolar transistor with nonlinear (both polynomial and piecewise-linear) backward trans-conductance. The derived and analyzed mathematical model is an autonomous third-order deterministic dynamical system. By considering the large six-dimensional hyperspace of internal system parameters dedicated to the searching-for-chaos routine, up to eleven different configurations of values turned out to be chaotic. The existence of strange attractors was proven via standard numerical algorithms such as surface-contour plots of the largest Lyapunov exponents. Geometrical structures of the typical chaotic attractors were quantified using the capacity and Kaplan–Yorke dimension and by calculating the approximate entropy using a generated time sequence. The long-term structural stability of numerically integrated chaotic attractors was confirmed by the construction of a flow-equivalent chaotic oscillator and experimental measurements. The captured oscilloscope screenshots are in good agreement with the theory. Although the bias point that characterizes the admittance parameters of the transistor model is hypothetical and probably unreachable in real life amplifier application, the discovered chaotic systems can be marked as novel due to their many interestingly shaped strange attractors.

There are many analog functional blocks that take advantage of the production of signals with increased entropy. True random bits generators (TRNGs) are examples of such electronic systems. Stoller et al. [2] introduce a novel TRNG and compare the randomness of its output signals with two other existing TRNG structures using tests established by the National Institute of Standards and Technology. A common feature of each investigated TRNG is the presence of W-SDC memristors as integrated circuits (developed and fabricated by Knowm Inc., Santa Fe, NM, USA). Memristors are key circuit elements and a source of the entropic properties of output data sequences. In the first TRNG, using Jiang´s topology, entropy is captured within a single memristor device during the transition time between its on and off state (represented by its low and high resistance, respectively). The same principle, but with the use of two memristors, is adopted in Rai’s TRNG structure. The final network, designed by the authors of this paper and termed the S-TRNG system, utilizes the coupling of two memristor-based multivibrators, the first with a high-speed and the second with a low-speed operational regime, and a comparator. Statistical tests based on the real measurements of sequences of 10^6^ values exhibit very good results for each constructed TRNG.

In many real-life situations, situations, mathematical model that describes analyzed physical object is not known exactly and only limited sequences of the measured/recorded data are available. Observed data are usually composed of combinations of multiple sources, both stochastic and deterministic. The so-called change-points detection problem (in the sense of the signal generating mechanism) within the dynamics of arbitrary nature is addressed in the comprehensive study of Piryatinska et al. [3]. The algorithm proposed by the authors follows the relatively new theory of the ε-complexity of continuous vector functions. Analyzed time series undergo the following detection procedure: choose the size of the sliding window, calculate the complexity coefficients for each window using the least means square method, repeat this process for each component of the sequence of complexity coefficients, and obtain estimates of the moments of change in the generation mechanism as a combination of detected change-points from both components of the complexity coefficients. It is useful for the retrospective analysis of observed data and can be used for the segmentation of data into homogenous fragments. Created routine can be considered as an alternative to other methods that are independent of the model of observed processes and is capable of quantifying the complexity of an analyzed data sequence. The proposed numerical algorithm suffers due to the small datasets of the multivariate data. However, this problem is recognized in other numerical methods as well.

Due to the general nature of such research, mathematical modeling and the theory of differential equations are sources of continuous new ideas and results. In Akhmet et al.’s study [4], the so-called modulo periodic Poisson stable (MPPS) functions, which are understood as a combination of the periodic and the Poisson stable functions, are discussed. The authors thoroughly investigate quasilinear differential equations that contain periodic and Poisson stable coefficients, which is quite rare in the available literature. The numerical simulation of MPPS functions and corresponding system solutions validates the theoretical results, as the authors demonstrate via several practical examples. From the perspective of future research, the analyzed MPPS functions can be used for the theoretical analysis of differential and discrete equations of various types. From the viewpoint of potentially chaotic systems (Lorenz, Rössler, Chua, etc.), it is of great interest to search for Poisson stability and its periodic components. Additionally, the achieved results can be applied to problems of optimization.

Biological systems are the subject of complex motions with ergodic properties if enough degrees of freedom are used to describe their current states. Choudhary et al. [5] introduce the concept of combination difference projective synchronization (CDPS) using third order generalized Lotka–Volterra (GLV) systems, i.e., a set of differential equations dedicated to describing the autonomous interaction of three species. The CDPS scheme is attained by designing appropriate nonlinear controllers on the basis of Lyapunov stability theory. Numerical simulations in Matlab show that a good choice of control function leads to the efficient asymptotical stabilization of a chaotic regime of GLV systems. The time demands for error functions to converge to zero are compared with other synchronization techniques with significant success. Three nonrealistic but chaotic GLV systems are involved, two working as masters and one as a slave. Besides the elaborated mathematical background focused on CDPS, the authors propose a block diagram of a new secure communication system. A chaotic signal is applied for message masking and as a recovery waveform. Two chaotic masters are employed to improve the protection of secure communication channels.

Social and economic dynamical systems are of significant complexity even if considered at a high level of abstraction. Nikolova et al. [6] proposed the extension of the well-known Dendrinos second-order autonomous mathematical model that describes the dynamics of ties between the public and private sector in general social systems. The added differential equation represents time changes caused by non-governmental organizations. After that, the analyzed dynamical system is smooth and contains only polynomial terms and four parameters. It is demonstrated that with increased system order, three dynamics can be long-term unpredictable and chaotic. The presence of Shilnikov’s kind of chaos is proven by the numerical findings of the homoclinic orbit. In this juicy paper, the study of a simplified social system is transformed into a qualitative analysis of a potentially chaotic system, where the appearance or disappearance of the equilibrium points, changes in the associated eigenvalues, and overall complexity of numerically integrated dynamic motion explain the influence between public, private, and non-governmental organizations.

The list of papers published in this Special Issue concludes with a contribution by Gonzalez-Diaz et al. [7]. Unlike the studies mentioned above, it is a more philosophical study focused on predictive sequential research design for complex social phenomena. It is pointed out that a single kind of study may be insufficient for reaching acceptable conclusions in this area, especially if the social phenomenon interacts with other entities. The idea described by the authors in this paper can be considered a proposal that has to be validated in the near future through its application in different fields of social and humanistic sciences. However, as is stated here, predictive analytics shows high accuracy in forecasting eventualities and scenarios, with fuzzy logic being the most effective method in data learning. This paper forms a nice ending to this Special Issue.

Of course, the seven papers published within this Special Issue address only a very small number of problems and difficulties that appear if nonlinear dynamical systems or time series need to be investigated. In addition to the issues currently being worked on worldwide, many other questions remain unanswered or hidden. There is strong reason to believe that the necessity of research aimed at nonlinear dynamics, chaos, flow quantification, mathematical modeling, and solutions to specific differential equations will lead to the emergence of similar Special Issues in the near future. I am looking forward to the upcoming developments in this wide scientific area that connects (through the universality of the mathematical apparatus) all fields of human life.
